# Design of a randomized trial to evaluate the influence of mobile phone reminders on adherence to first line antiretroviral treatment in South India - the HIVIND study protocol

**DOI:** 10.1186/1471-2288-10-25

**Published:** 2010-03-26

**Authors:** Ayesha De Costa, Anita Shet, Nagalingeswaran Kumarasamy, Per Ashorn, Bo Eriksson, Lennart Bogg, Vinod K Diwan, the HIVIND study team

**Affiliations:** 1 Division of Global Health, Nobels Väg 9, Karolinska Institutet, 171 77 Stockholm, Sweden; 2 St Johns National Academy of Health Sciences, Sarjapura Road, Bangalore 560034, India; 3 YR Gaitonde Centre for AIDS Research and Education, VHS Campus, Rajiv Gandhi Road, Taramani, Chennai 600113 India; 4 Medical School, University of Tampere, ARVO building, Tampere, Finland 33014; 5 Cavidi AB, Uppsala Science Park 751 83 Uppsala, Sweden

## Abstract

**Background:**

Poor adherence to antiretroviral treatment has been a public health challenge associated with the treatment of HIV. Although different adherence-supporting interventions have been reported, their long term feasibility in low income settings remains uncertain. Thus, there is a need to explore sustainable contextual adherence aids in such settings, and to test these using rigorous scientific designs. The current ubiquity of mobile phones in many resource-constrained settings, make it a contextually appropriate and relatively low cost means of supporting adherence. In India, mobile phones have wide usage and acceptability and are potentially feasible tools for enhancing adherence to medications. This paper presents the study protocol for a trial, to evaluate the influence of mobile phone reminders on adherence to first-line antiretroviral treatment in South India.

**Methods/Design:**

600 treatment naïve patients eligible for first-line treatment as per the national antiretroviral treatment guidelines will be recruited into the trial at two clinics in South India. Patients will be randomized into control and intervention arms. The control arm will receive the standard of care; the intervention arm will receive the standard of care plus mobile phone reminders. Each reminder will take the form of an automated call and a picture message. Reminders will be delivered once a week, at a time chosen by the patient. Patients will be followed up for 24 months or till the primary outcome i.e. virological failure, is reached, whichever is earlier. Self-reported adherence is a secondary outcome. Analysis is by intention-to-treat. A cost-effectiveness study of the intervention will also be carried out.

**Discussion:**

Stepping up telecommunications technology in resource-limited healthcare settings is a priority of the World Health Organization. The trial will evaluate if the use of mobile phone reminders can influence adherence to first-line antiretrovirals in an Indian context.

**Trial Registration:**

Trial registration: ISRCTN79261738.

## Background

In the current era of enhanced access to antiretroviral treatment (ART) globally, the number of people on ART in low and middle income countries by the end of 2008 has crossed the 4 million mark, with an increase of 1 million from the end of 2007[1]. However, one of the greatest challenges associated with the management of HIV is suboptimal adherence to antiretroviral therapy [2]. Good adherence to ART is beneficial to patients, as it minimizes treatment failure and prolongs survival. In addition, it has public health benefits. This is because poor adherence gives rise to the potential for development of drug-resistant strains [3], necessitating administration of expensive second-line therapy, and the possibility of transmission of drug-resistant HIV by non-adherent patients [4,5].

Adherence is known to be influenced by diverse characteristics of individuals, by their environments and by the treatment regimen prescribed [6]. There have been some successful interventions targeting patient-related social and psychological barriers to adherence, including Directly Administered ART (DAART) and peer support [7,8]. However, concerns have been raised over the quantum of healthcare resources that may be required to implement and sustain such expensive interventions, particularly in resource-constrained settings [6]. The Cochrane database of systematic reviews [9], reviewed studies of patient support and education for promoting adherence to ART. The 10-year review (1996-2005) included only studies, which used a randomized controlled trial design to assess the intervention and measured adherence at a minimum of 6 weeks. Only 19 studies met these inclusion criteria, and none of these had been conducted in low income settings (12 were in the US, 5 in Europe and 2 in Australia). Thus there is a need, particularly in low-income settings, to use sustainable contextual adherence aids, and to test these aids, using rigorous scientific design, at the population level.

There has been recent interest in the potential use of mobile phone technology for various aspects of health care, particularly in low-income settings [10-12]. Presently, 64% of all mobile phone users are in the developing world [13]. The current ubiquity of the mobile phone in resource-constrained settings [14], makes phone contacts a contextually appropriate, relatively low-cost means of supporting adherence. There have been few reports on the use of mobile phones in HIV care, within small cohorts of HIV patients from North [15]and South America [10], and Africa [16]. There have, so far, been no reports from randomized trials evaluating the influence of mobile phone reminders on adherence to ART; however a Kenyan trial evaluating this concept is currently ongoing [17].

India, which has over 275,000 patients on first line ART[18], is well suited to explore the influence of the mobile phone on adherence to ART, given the widespread connectivity, low costs and growing popularity of the mobile phone. Between 10 and 12 million connections are added every month to a base of 471 million connections [19]. Given that India is scaling up its national program for access to first-line ART, and that the use of the mobile phone is so widespread and relatively inexpensive, we plan to test in randomized controlled trial design, a hypothesis that mobile telephone reminders will influence adherence and hence time to virological failure in ART naïve, HIV infected patients, initiated on first-line therapy in South India. This protocol describes the methods proposed in the conduct of the trial.

## Methods/Design

This study is a two-centre, parallel group, randomized controlled trial. Eligible participants are randomized in a 1:1 allocation ratio to one of two arms: an intervention arm, in which the participants receive reminders via their mobile phones in addition to the standard of care(defined below); and a control arm in which they receive only the standard of care.

### Study setting and participants

Patients will be recruited from two institutions located in the capital cities of two different provinces in South India. One of these is an infectious disease clinic at a teaching university hospital in Bangalore, while the other is a comprehensive HIV treatment and care center in Chennai. Both are non-profit organizations with large clinics catering to both urban and rural populations in South India.

#### Inclusion criteria

Eligible participants include (i) HIV positive patients, (ii) between 18-60 years of age, (iii) who are ART naïve (defined as not having taken ART for more than 14 consecutive days previously),(iv) reside in the South Indian provinces, (v) and who meet criteria for initiation of first-line ART as per the national guidelines [20].

#### Exclusion criteria

Patients who are (i) severely ill (Karnofsky score[21] less than 70), (ii) those who are unable to participate in all study visits or (iii) those who reside in an area where there is no mobile telephone network will not be eligible to participate in the study. Further, to prevent contamination effects, patients belonging to a household where another member has already been recruited into the study, will not be eligible.

#### Informed consent

All eligible patients will individually be given an initial verbal description of the proposed study by a research assistant. Interested individuals will then be presented a written informed consent form. The form will also be verbally explained as there likely to be varying levels of literacy among potential participants. A signature, or thumb imprint, will be obtained from all those who consent to participate. The written informed consent form will be in English, Kannada and Tamil, as patients attending the study clinics speak one or more of these languages. Consent will be obtained by a trained research assistant, who is not involved in the routine clinical care of the patient.

### Randomization

Stratified randomization, separately for male and female participants, will be performed. Within each stratum, block randomization will be used to ensure balanced representation in the two treatment arms (control and intervention) as recruitment progresses. Blocks of 4 and 6 will be used. Sequentially numbered opaque sealed envelopes will serve as an allocation concealment method [22]. Envelopes will be located at the two study clinics. Once randomized, patients will be assigned to their respective study arms by a study nurse, and each will be given a study identification number.

### Intervention

The intervention which will be evaluated in this trial will consist of interactive voice and text messages sent to patients' mobile phones as reminders to take their ART. This intervention will be compared against the standard of care.

#### Standard of care

All patients in the study will receive the routine standard of care, as prescribed by the Indian national ART guidelines[18]. This will include three counseling sessions prior to initiation of ART, routine clinical and laboratory tests at baseline, and follow-up assessments as prescribed in the guidelines.

The intervention was designed following several discussions with the project team and with non-project clinical staff, as well as a pilot interview study with patients routinely attending the clinics [23].

The pilot study revealed that although 75% of patients owned mobile phones, use of the text messaging function was low, possibly because of relatively low literacy rates. Phones were used mostly for conversation. For the study intervention, an automated interactive voice message in the local language was selected, along with neutral pictorial messages instead of text messages, as these are language-independent and are less likely to lead to stigma should the phone be shared.

Thus the intervention will consist of 2 parts (i) an a *utomated voice call *that will go out once a week to patients in the intervention arm, at a time decided upon by each patient. The call will be interactive, as the patient will respond to a question about whether his or her pill doses were taken the previous day, by pressing '1' for yes or '2' for no. If the patient fails to respond to the call, a maximum of three more calls will be made over the ensuing 24 hours, until a response is obtained. (ii) A weekly non interactive neutral pictorial message will be sent out as a reminder 4 days after automated call.

All participants in the intervention arm will receive an explanation of the details of the intervention at recruitment. Participants will hear the recorded message that they will receive as an automated call, and informed of how to respond to this (clicking the digits 1 or 2 as appropriate). In addition, participants will also be trained on how to view messages on their phones and shown the image that they will receive as a reminder.

Patients in the control arm will also be given a mobile phone, but no interventions as described above will be made in this arm. This will be done to obviate any effect that simply 'receiving a mobile phone' might have on adherence.

Participants will receive the intervention at no added cost, as receiving calls or text messages are free within Indian phone networks. All participants (control and intervention) will be given air time credit of Rs. 100 (US$ 2.5) per month. Participants in both arms can call the clinic staff via their mobile phones whenever necessary, and a record of these calls will be maintained in a log book.

### Outcome Measures

#### Primary outcome

The primary outcome will be measured as time (in weeks) to virological failure which defined as a plasma viral load of more than 400 copies/ml on each of two consecutive samples measured one month apart, six months after initiating ART. The date of the first assessment of a plasma viral load more than 400 copies/ml will be documented as the date of virological failure. Virological failure was chosen as a primary outcome, as this is a biological surrogate marker of poor adherence, and is an objective measure. For the primary outcome, patients in each arm will be followed up for 24 months (after initiating ART) or until virological failure occurs, whichever is earlier.

#### Secondary outcome

Participant adherence to ART during the study period of 24 months will be the secondary outcome. At each visit, adherence is measured using the 4-day recall [24] and the 30-day visual analogue scale [25]. A pill count is also done at every follow-up study visit.

### Quality Control

Standard operating procedures for key processes in the study have been developed. Prior to recruitment, the field team will receive extensive training on the objectives, methods and processes of the study, as well as on the concept of ethical research. In order to achieve optimal quality of data from questionnaires and laboratory tests, training and random checks by the site coordinator will be adopted. Completed questionnaires will be reviewed daily by the site coordinator for completeness, errors and inconsistencies. Quality assurance protocols will be set in place to maintain quality of processes and data collection in the clinic. In addition data collected will be subject to a second phase of checking through in built checks in the database (while entering data) and supervision of data entry by a trained person.

### Sample size

The primary end point in the trial will be time to virological failure as estimated using the standard life-table technique and illustrated by Kaplan Meier survival curves [26]. The baseline assumptions in the sample size calculation, are that the risk for virological failure in the control arm will be 10% [27] (risk rate 0.1), while the intervention will reduce this risk to 3% (0.03). The assumptions for this level of risk in the control arm and intervention arm are based on a review of available literature [8,28,29]. Stata 10 sample size software module was used to determine the necessary sample size for a comparison of the survival times between the two arms, using the log rank two-sided test. The significance level or alpha level was set to 0.05 for a two-tailed test, and the power (probability to detect a deviation of hazard ratio from unity) requested was 0.90 (90%). Given this, a sample size of 266 in each arm (n = 1:1) was determined. Assuming an attrition rate of 10%, this sample size was inflated by 10% to compensate for loss to follow-up, which gives a total sample of 292 in each arm. Thus a total of 600 patients newly initiated on ART will be recruited, with 300 in each arm of the trial. By having 300 participants in each arm, the trial will have sufficient power to detect a clinically significant difference between the arms. With a planned recruitment rate of 4-8 participants per week at each centre, the recruitments for the study will be completed within approximately 12 to18 months.

**Figure 1:**
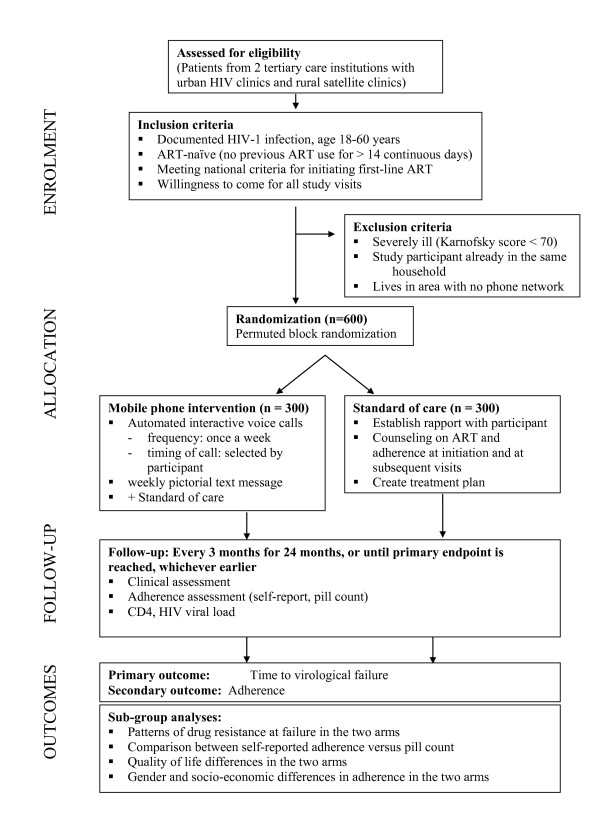
**Flow Diagram of Study Design**.

A flow diagram of the study design is presented in figure 1.

### Statistical analysis

The primary aim of the statistical analysis is to compare the two arms, with respect to the primary and secondary outcomes, i.e. the time to virological treatment failure and adherence. In both cases, we plan to estimate the deviation between the two arms and assess statistical significance, i.e. the probability that the deviation is due to random variation. The estimation results will be given as point estimates and confidence intervals.

Given the primary end point, the conventional approach for the analysis of risks would be to study the risk ratio and hazard ratio (RR, HR). In addition, the study of the risk difference (RD) will provide information on the expected number of cases that actually benefit from the intervention in terms of avoided failures.

The risk analysis will be carried out using survival analysis techniques. Survival curves will be constructed using the Kaplan-Meier life table technique; these will then be compared using log- rank tests, possibly stratified. Cox regression models will also be employed. As the risks of treatment failure are likely to be rather small, a simplified analysis, using for e.g. Poisson regression will give very similar results. The observed time for any patient will be adjusted for periods that could not be observed due to no-show. Patients withdrawn for reasons other than virological failure, and patients lost to follow-up, will be right censored, but included in the analysis for the periods during which they could be observed.

With regard to the secondary outcome, the different adherence variables will be numeric hence other models and tests are required. The main aims are to study the average adherence, variation of adherence within as well as between participants, and trends over time. Increased adherence might be of limited value if the variation remains large. Different models for Analysis of Variance (ANOVA) with provision for repeated measurements will be used. The distribution of adherence at any point in time is likely to be negatively skewed. This will necessitate analysis, using distribution-free (non-parametric) statistical methods, or transformations of original data.

Intention to treat analyses (ITT) will be used for the comparison of primary and secondary outcomes between arms. Thus a patient assigned to the intervention arm will be considered as belonging to that arm even if he or she clearly does not perform according to the protocol.

Analyses will be carried out using the program STATA, version 10.

#### Interim analysis

An independent data safety and monitoring committee, including a statistician, has been established to perform an interim analysis. This analysis of the failures occurring in each arm is scheduled to occur when 150 patients have been recruited into each arm. The interim analysis team will carry out the analysis blinded. The primary aim of the interim analysis is to assess if the intervention arm shows a clearly better, or worse, outcome, in terms of failures, than the control arm. The result will guide the discussion about whether to continue or end the trial. The interim analysis will also consider the accumulated reported adverse effects in both two arms.

#### Subgroup analyses

In addition to analyses that aim at hypothesis testing, we will also carry out exploratory analyses that will produce descriptive data that can be used for subsequent hypothesis generation and to provide additional information about the effects of the intervention on different participant sub groups (by age, sex, rural/urban, socioeconomic strata, literacy).

### Other sub-studies

The trial will also provide a clinical platform to study adverse drug reactions to national first-line ART among newly ART-initiated individuals. Other clinical studies will include those on the incidence of opportunistic infections, on immune reconstitution inflammatory syndromes, and on the influence of ART on changes in nutritional status and dietary intake, within two years of initiating ART. In addition, in the intervention arm, adherence reported over the telephone will be compared with self reported measures. Qualitative studies exploring the responses and perceptions of intervention arm participants towards the mobile phone intervention and the experiences of clinic staff with this modality will be included.

### Economic analysis

A cost-effectiveness analysis of the intervention from a governmental perspective will be performed. The principle of marginal costing will be used to cost the resources utilised in the alternative strategies. Full costing, by absorption costing or by activity based costing, would not be relevant for this study, since the purpose is to provide a basis for rational decision making. In a decision-making process the fixed costs are irrelevant, since they will not be affected by the choice of either alternative strategy. All costs of a historical nature, so-called sunk costs such as resources invested in buildings, equipment, and vehicles will be ignored. Only costs related to the future, from the onset of the study, will be recorded.

A further restriction is that only differential costs will be collected, i.e. costs that are different between the two alternative strategies. Costs that are common to the two alternative strategies will be ignored. In the case of use of materials, the replacement cost will be used, i.e. not the accounting cost or the historical cost of the resources, but the current cost of replacing the resources will be applied.

The process of costing will be as follows. First, the relevant resources will be identified that are differential for the alternative strategies. Second, the quantities of resource use will be measured, e g the time spent by different clinic staff on the patient. Third, the cost of the resource consumption will be calculated, by multiplying the time or numbers of the resource with a standard cost of the resource. The historical prices recorded in the accounts will not be used, since these would result in measuring differences in price levels rather than differences in resource consumption.

### Ethical issues

The study protocol has been ethically approved by institutional ethical review boards in all the participating and collaborating institutions. The rights and welfare of the participants will be protected according to the Declaration of Helsinki Ethical Principles for Medical Research Involving Human Subjects. Clinical care and emergency medical services are provided by the participating institutions. Data that is collected as part of the study will be not be linked to any individual, personal identifiers will not be used in data storage, and confidentiality will be maintained at all levels of data management.

An important consideration in this study design is the possibility of stigma, as patients may not have disclosed their HIV status to people they live and work with. It is conceivable that some stigma may be attached to receiving a mobile phone from the hospital. Participants in the intervention arm also run the risk of being stigmatized, due to the receipt of regular text and voice messages. To minimize this risk, the picture messages that the patients will receive as reminders, will be neutral pictures, which the patient is shown, and approves at the enrollment visit. With regard to the automated call, the patient chooses a particular time based on his schedule, when s/he has the privacy to receive and respond to the call. Detailed counseling on potential stigma issues will accompany routine counseling at recruitment and at subsequent study visits.

## Discussion

### Methodological

As patients are not blinded to the intervention they receive, a Hawthorne effect in the intervention arm is a possibility. This could exaggerate the difference in adherence between the two arms. Also the attention given to trial participants at the clinics, in itself may raise adherence in both arms.

With regard to the intervention, the receipt of automated calls can be recorded, hence the study team will know that the call was received and attended to (based on the response received from the patient). However, though patients will be trained to look at the sms on their phone, it is not possible to know if the patient actually read the sms sent out, or if it remained unread on his/her phone. Interactive sms alone was not chosen as a method in this study, given the low usage of the sms function in this setting [23].

The trial will study the influence of the entire intervention. It will not be able to assess the degree to which each component of the intervention contributed to any observable difference.

### Telecommunications in healthcare in resource limited settings

The World Health Organization has indicated that incorporating the use of newer technology, such as telecommunications, in resource-limited settings to enhance healthcare delivery, is a priority [30]. Mobile phones offer promise for use in healthcare, particularly in low income settings, given the centrality of their use in everyday life. Living in resource-poor environments is not a barrier to the use of mobile phones. There is evidence that the existence of a so-called "digital divide" along the socio-economic gradient is less pronounced when it comes to mobile phones than with regard to other communication technologies, such as the Internet [14].

The telecommunications network in India is the third largest in the world, with a total of 509 million connections, of which 471 million are mobile telephone connections [31]. The use of mobile phones in the country has increased rapidly along with falling costs of mobile telephone services. India has also begun to scale up of it's National AIDS control program, providing first-line ART free of cost. In a pilot study at one of the trial centers clinic [23], we found that 75% of clinic attendees owned mobile phones, making this a contextually appropriate intervention.

Though studies on the use of telephones in promoting adherence to ART exist [28,32], they have been carried in high-income areas of the world, where fixed telephone infrastructure exists. These trials reported no influence on time to virological failure, but did indicate a positive impact on self-reported adherence.

Mobile telephones are increasingly relevant in India, and in many parts of the developing world, as they are low-cost, easily available and used commonly in everyday life [33]. The results of this trial are expected to provide evidence of the influence of mobile phone reminders on improving adherence to first-line ART in an Indian context. This intervention is also expected to delay treatment failure, and hence preserve expensive second-line ART. As this is the first such trial in India as well as in Asia, the results will highlight key implications for other low-income settings globally, where there is rapid growth in acess to mobile phones, and the potential for their incorporation into many aspects of routine healthcare.

## Competing interests

The authors declare that they have no competing interests.

## Authors' contributions

ADC drafted the manuscript. ADC and VKD wrote the original protocol. AS, NK, CK and PA, were co-applicants on the grant application and also actively contributed to study design and to the refined the protocol. ADC, AS, NK, CK and PA also worked with the ethics applications at the sites. BE and KA contributed to the statistical plan and LB contributed to the cost effectiveness study design. AS, ADC and AKG helped with organizing and coordinating the phone intervention. UN, RR, ML, VK, SK and GDS participated in study design and ethics applications. UN also worked with the laboratory coordination. AS, VK, NK, GDS and PBS provided valuable clinical expertise while designing and refining the protocol. All authors read and approved the final manuscript.

## Pre-publication history

The pre-publication history for this paper can be accessed here:

http://www.biomedcentral.com/1471-2288/10/25/prepub
